# Combining topical and intravenous tranexamic acid in cardiac surgery: does it really matter? – a systematic review and meta-analysis

**DOI:** 10.1016/j.ijcha.2025.101848

**Published:** 2025-12-01

**Authors:** Paul C. Onyeji, Leo Consoli, Amrinder Kaur, Shivank Dani, Sonise Momplaisir-Onyeji, Felipe S. Passos, Hristo Kirov, Torsten Doenst, Tulio Caldonazo

**Affiliations:** a All Saints University School of Medicine, Roseau, Commonwealth of Dominica; b Faculty of Medicine, Federal University of Bahia, Salvador, Brazil; c The University of Chicago, Chicago, IL, United States; d GMERS Medical College and Hospital, Sola, Ahmedabad, India; e American University of Barbados, Wildey, Barbados; f Department of Thoracic Surgery, MaterDei Hospital, Salvador, Brazil; g Department of Cardiothoracic Surgery, Jena University Hospital, Jena, Germany; h Department of Cardiothoracic Surgery, Weil Cornell Medicine, NY, United States

**Keywords:** Intravenous, Topical, Tranexamic acid, Cardiac surgery

## Abstract

**Background:**

The benefit-to-risk ratio of administration of intravenous (IV) and topical tranexamic acid (TXA) together in cardiac surgery has not yet been determined. This study aims to evaluate whether the combined approach (IV plus topical TXA) offers superior bleeding control compared to IV TXA alone, while maintaining an acceptable safety profile.

**Methods:**

We conducted a systematic review and *meta*-analysis of randomized controlled trials (RCTs) and observational studies comparing combined topical and intravenous TXA administration versus IV TXA alone in patients undergoing cardiac surgery. The primary outcome was cumulative blood loss. Secondary outcomes included all-cause mortality, transfusion-free status, and the number of transfused blood products. A random-effects model was used for all pooled analyses.

**Results:**

We included a total of five studies (four RCTs, one observational; n = 880). Pooled analysis showed that the combined approach significantly, but modest, reduced total blood loss when compared to an IV-only TXA strategy (MD −39.84, 95 %CI −74.80 to −4.88; p = 0.03; I^2^ = 39 %). However, this benefit did not translate into a significant reduction in transfusion requirements (OR 1.00, 95 %CI 0.72 to 1.37; p = 0.98; I^2^ = 0 %), volume of blood products used (MD −0.01, 95 %CI −0.04 to 0.02; p = 0.51; I^2^ = 0 %), or all-cause mortality (OR 0.85, 95 %CI 0.24 to 3.08; p = 0.81; I^2^ = 0 %).

**Conclusion:**

Combined topical and IV TXA application is associated with reduced total blood loss after cardiac surgery compared to an IV-only approach. However, no significant differences were observed in transfusion rates, blood product utilization, or mortality.

## 1 Introduction

Tranexamic acid (TXA), a potent antifibrinolytic agent, is a cornerstone for managing postoperative bleeding in cardiac surgery. Although its systemic intravenous (IV) administration is endorsed with a Class 1A recommendation by major societies, including the Society of Thoracic Surgeons, the Society of Cardiovascular Anaesthesiologists, and the International Society for Minimally Invasive Cardiothoracic Surgery [1,2], postoperative bleeding and transfusion remain frequent and clinically significant complications. The optimal strategy to enhance hemostasis while minimizing adverse events is still under investigation.

While systemic TXA effectively reduces blood loss, high doses have been associated with increased risk of postoperative seizures, without offering additional hemostatic benefit. A 2019 *meta*-analysis by Guo et al. [3] underscored this concern, encouraging the exploration of alternative or adjunctive delivery routes. Among them, topical TXA has gained interest for its ability to provide local hemostasis with limited systemic absorption. Preliminary studies suggest it may reduce bleeding and re-exploration rates [[4], [5], [6], [7]], leading to growing interest in combining systemic and topical TXA.

Despite its theoretical appeal, the efficacy and safety of this combined approach remain uncertain. Evidence from individual studies is inconsistent, some report improved outcomes, while others show no benefit or even higher transfusion needs. To address this clinical equipoise, we conducted a systematic review and *meta*-analysis comparing combined topical and systemic TXA with systemic TXA alone in adult patients undergoing cardiac surgery.

## 2 Methods

The study selection followed the Preferred Reporting Items for Systematic Reviews and Meta-Analyses (PRISMA) guidelines [8,9]. The review was registered in the International Prospective Register of Systematic Reviews (PROSPERO, ID CRD42024572330).

### 2.1 Search strategy

A comprehensive literature search was performed on Ovid MEDLINE, EMBASE and the Cochrane Library to identify studies comparing outcomes between combined topical and IV versus IV-only TXA application in cardiac surgery, published up to June 2025. Additionally, we searched for additional studies using the references of previously included studies. The complete search strategy is available in Supplementary Table S1.

### 2.2 Study selection

After removing duplicates, two independent reviewers (PO and AK) screened titles and abstracts. Selected studies were then reviewed in full using the inclusion and exclusion criteria. Any discrepancies and disagreements were resolved by a third author (FP).

### 2.3 Eligibility criteria

Studies were included in this *meta*-analysis if they met the following criteria: 1) randomized controlled trials (RCTs) or comparative observational studies; 2) included patients undergoing cardiac surgery; 3) directly compared the use of combined IV and topical TXA versus IV-only TXA; and 4) were full-text articles published in English. Studies were excluded if they were case reports, case series, conference abstracts, animal studies, or non-comparative in design.

### 2.4 Quality assessment and publication bias

The quality of included studies was assessed by two authors (LC and SD) using the Cochrane Collaboration tool for assessing the risk of bias in non-randomized studies (ROBINS-I) tool for observational studies and the Risk of Bias 2 (RoB 2) for RCTs (Supplementary Fig. S3). Publication bias was assessed for the primary outcome.

### 2.5 Data extraction and baseline characteristics

Two authors (PO and SM) independently performed data extraction. Discrepancies were resolved by consensus. The extracted variables included study characteristics (author, year of publication, country, and sample size), baseline patient data (mean age, sex, weight, preoperative left ventricular ejection fraction, fibrinogen, hemoglobin, hematocrit, platelet count, and international normalized ratio), and outcome data.

### 2.6 Outcomes

The primary outcome was cumulative blood loss. Secondary outcomes included transfusion-free patients, all-cause mortality and transfused blood products.

### 2.7 Statistical analysis

Odds ratios (OR) with 95 % confidence intervals (CI) were calculated for binary outcomes and mean differences (MD) were calculated for continuous endpoints. Cochran Q test and I^2^ statistics were used to assess heterogeneity; P values inferior to 0.10 and I^2^ > 25 % were considered significant for heterogeneity. A p-value < 0.05 was considered statistically significant. DerSimonian–Laird random effects models were used for all endpoints. The Cochrane Handbook for Systematic Reviews of Interventions was used for data handling and conversion. To ensure robustness of our findings, a leave-one-out analysis was performed. All statistical analyses were performed using R (version 4.4.0, R Foundation for Statistical Computing, Vienna, Austria).

## 3 Results

### 3.1 Study characteristics

**Fig. 1:**
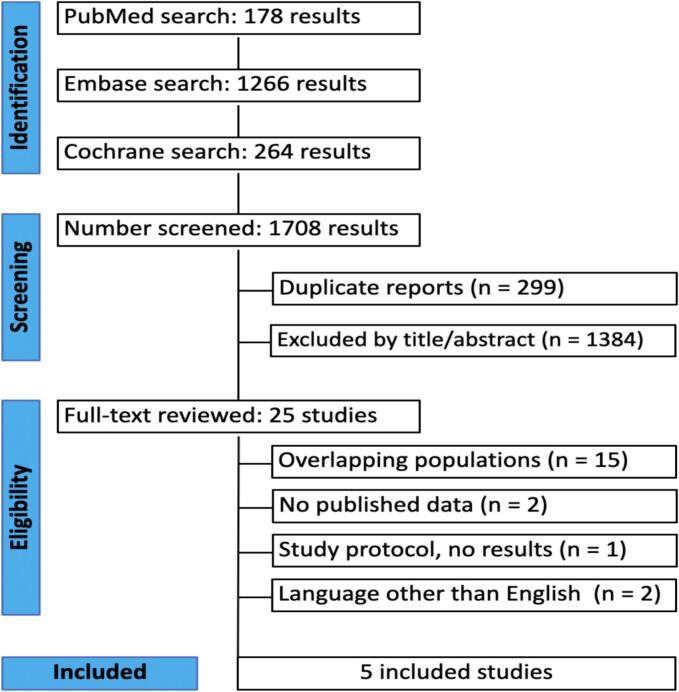
Preferred Reporting Items for Systematic Reviews and Meta-Analyses (PRISMA) flow diagram.

Fig. 1 shows the PRISMA flow diagram outlining the study selection process. The search strategy identified 1,708 results. After deduplication and exclusion based on title and abstract, 25 studies remained for full-text review. Of these, five studies met all the inclusion criteria for the analysis [[10], [11], [12], [13], [14]].

### 3.2 Patient characteristics

Table 1 shows the individual study information. Four RCTs and one observational study were included in this *meta*-analysis, encompassing 880 patients. Among these, 439 patients received combined topical and IV TXA application, while 441 received IV-only. The number of patients in each study ranged from 50 to 490. The age ranged from 8 to 76 years, with the percentage of male patients varying from 42 % to 82 %.

**Table 1:** Baseline characteristics of the included studies.

**Study**	**Design**	**Country**	**Sample size** **C/IV**	**Age Mean, y** **C/IV**	**Weight (kg)** **C/IV**	**Male, n** **C/IV**	**Pre-op LVEF(%)** **C/IV**	**Pre-op FIB** **C/IV**	**Pre-op HGB** **C/IV**	**Pre-op HCT** **C/IV**	**Pre-op PLT** **C/IV**	**Pre-op INR** **C/IV**
**Taksoudum 2017**	RCT	Thailand	40/ 40	70/ 71	N/A	17/ 18	60.8/ 57.9	299.0/ 318.9	13.4/ 12.7	38.4/ 39.0	207.6/ 239.9	1.1/ 1.0
**Kimenai 2016**	RCT	Netherlands	245/ 245	75/ 76	84/ 84	196/ 180	N/A	3.4/ 3.4	14.0/ 13.8	41/ 40	241/ 244	1.0/ 1.0
**Spegar 2011**	RCT	Czech Republic	49/ 51	73/ 74	81.7/ 80.0	28/ 26	52.3/ 55.0	4.25/ 3.93	14.0/ 13.8	41.44/ 40.40	216.1/ 217.1	1.11/ 1.10
**Mahaffey 2013**	Observational	Canada	80/ 80	75/ 75	86.7/ 87.0	61/ 64	N/A	N/A	13.2/ 13.4	N/A	219.7/ 228.8	1.1/ 1.1
**Patel 2017**	RCT	India	25/ 25	11/ 9	14.95/ 12.83	20/ 17	N/A	N/A	16.50/ 15.38	N/A	320/ 310	1.20/ 1.14

*Mean or median; n: number; C: combined tranexamic acid group; IV: intravenous tranexamic acid group; LVEF: Left ventricular ejection fraction; TOP: Topical Tranexamic acid; IV: Intravenous Tranexamic acid; HGB: Hemoglobin; HCT: Hematocrit; INR: International normalised ratio; N/A: Not available; Pre-op: Preoperative. Continuous variables are mean ± SD in the case of normal distribution, otherwise reported as median (IQR). Categorical data are reported as *n* (%).

### 3.3 Outcomes

**Fig. 2:**
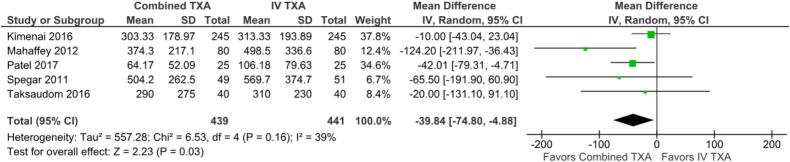
Forest plot for the outcome of cumulative blood loss, comparing the use of combined TXA versus IV alone. CI: confidence interval; IV: intravenous; SD: standard deviation; TXA: tranexamic acid.

Table 2 summarizes the *meta*-analysis findings. Combined TXA was associated with a significant, but modest, reduction in cumulative blood loss compared to IV only application (MD −39.84 ml; 95 % CI −74.80 to −4.88; p = 0.03; I^2^ = 39 %; Fig. 2).

**Table 2:** Summary of outcomes.

**Outcome**	**Number of Studies**	**Number of Patients**	**Effect Estimate, Random Model**
			**(95 % CI, p-value)**
Cumulative Blood Loss	5	880	MD −39.84, 95 % CI −74.80 to −4.88, p = 0.03, I^2^ = 39 %
Transfusion-free Patients	3	640	OR 1.00, 95 % CI 0.72 to 1.37; p = 0.98, I^2^ = 0 %
All-Cause Mortality	3	750	OR 0.85, 95 % CI 0.24 to 3.08; p = 0.82, I^2^ = 0 %
Blood Products Transfused	4	767	MD -0.01, 95 % CI −0.04 to 0.02; p = 0.51, I^2^ = 0 %

CI: confidence interval; MD: mean difference; OR: odds ratio.

A leave-one-out sensitivity analysis for this outcome revealed that the overall effect size lost statistical significance when the study by Patel et al. was excluded (Supplementary Fig. S1). The funnel plot did not reveal significant asymmetry on visual inspection (Supplementary Fig. S2). Quantitative analysis of asymmetry (i.e. Egger’s test) was not performed due to the limited number of studies included.

**Fig. 3:**
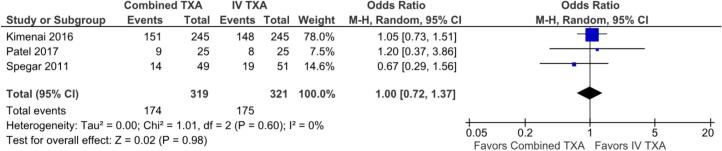
Forest plot for the outcome of transfusion-free patients, comparing use of combined TXA versus IV alone. CI: confidence interval; IV: intravenous; TXA: tranexamic acid.

**Fig. 4:**
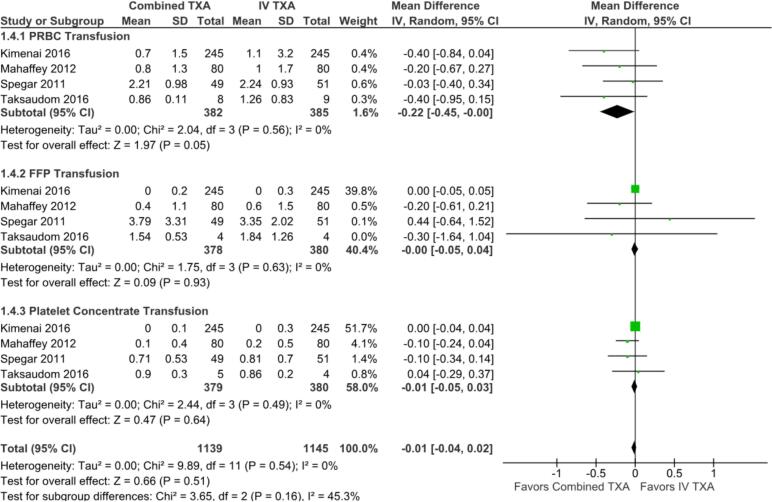
Forest plot of the outcome of blood products transfused, comparing combined TXA to IV alone. CI: confidence interval; FFP: fresh frozen plasma; IV: intravenous; PRBC: packed red blood cell; SD: standard deviation; TXA: tranexamic acid.

**Fig. 5:**
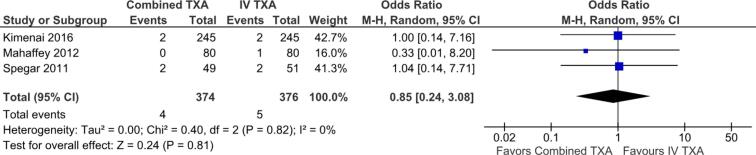
Forest plot for the outcome of all-cause mortality, comparing use of combined TXA versus IV alone. CI: confidence interval; IV: intravenous; TXA: tranexamic acid.

There was no significant difference between groups in secondary outcomes, including transfusion rates (OR 1.00; 95 % CI 0.72 to 1.37; p = 0.98; I^2^ = 0 %; Fig. 3), volume of transfused blood products (MD -0.01, 95 % CI −0.04 to 0.02; p = 0.51, I^2^ = 0 %; Fig. 4), and all-cause mortality (OR 0.85; 95 %CI 0.24 to 3.08; p = 0.82; I^2^ = 0 %; Fig. 5).

### 3.4 Quality assessment

The risk of bias was assessed using the RoB 2.0 tool for RCTs and ROBINS-I tool for the observational study (Supplementary Fig. S3). Mahaffey et al. [13], the only observational study, was judged to have an overall serious risk of bias, primarily due to concerns related to confounding. There was also moderate risk of bias in measurement of outcomes. For all other domains, this study was rated as having a low risk of bias.

Among RCTs, the three studies by Taksaudom et al. [10], Kimenai et al [11], and Spegar et al. [12] were evaluated as having low risk of bias. Meanwhile, Patel et al. [14] had some concerns in the randomization process. These findings suggest a generally high methodological quality among the included RCTs and acceptable quality in the observational study.

## 4 Discussion

In this systematic review and *meta*-analysis of four RCTs and one observational study including 880 patients, we comprehensively analyzed the outcomes of combined topical and IV TXA compared to IV administration alone in cardiac surgery. Our main findings were as follows: (I) combined approach is related to significant, but modest, reduction in cumulative blood loss; however, this association was lost in the leave-one-out sensitivity analysis; and (II) no significant differences were found in transfusion-free patients, volume of transfused blood products and all-cause mortality.

The impact of a combined TXA approach on postoperative bleeding showed notable variation across studies. Mahaffey et al. [13] demonstrated a 25 % reduction in 12-hour chest tube drainage in combined therapy compared to IV-only strategy. Similarly, Spegar et al. [12] observed lower 24-hour blood loss in the combined therapy group, though the difference in mean values did not reach statistical significance. However, they noted significantly reduced variance in blood loss patterns, suggesting more consistent hemostasis with the combined approach.

Conversely, Kimenai et al. [11] the largest randomized trial, and Taksaudom et al [10] found no significant difference in blood loss between combined and IV-only administration. This discrepancy may reflect differences in surgical techniques, patient populations, and institutional hemostatic protocols. Notably, Patel et al. [14], a pediatric study in cyanotic congenital heart disease, demonstrated the most pronounced benefit with combined therapy, suggesting improved efficacy in high-risk populations. However, in our *meta*-analysis, the reduction in blood loss did not retain statistical significance in the leave-one-out analysis after omission of the Patel et al. [14] study, indicating that this apparent effect was largely driven by that single trial.

These findings align with *meta*-analyses in other surgical fields. Xiong et al. [15] found that combined TXA administration reduced blood loss in knee arthroplasty, while Guo et al. [3] concluded that IV administration remains superior for hemostasis in cardiac surgery. Our results suggest a potential adjunctive role for topical TXA, particularly in high-bleeding-risk scenarios.

Blood product utilization patterns generally paralleled the observed trends in postoperative bleeding. In Mahaffey et al. [13] fewer patients in the combined group required packed red blood cells (29 vs. 33), fresh frozen plasma (11 vs. 15), and platelets (6 vs. 9), though these differences were not statistically significant. Spegar et al. [12] demonstrated a significant reduction in fresh frozen plasma use with combined therapy (42.9 % vs. 70.6 % of patients, p = 0.008).

Kimenai et al. [11] found no differences in blood product usage between groups, suggesting that in the context of a strict transfusion protocol with point-of-care testing, the route of TXA administration may be less critical. The pediatric study by Patel et al. [14] demonstrated the most pronounced benefit in blood product conservation with combined therapy. This finding carries particular significance in pediatric cardiac surgery, where exposure to multiple blood products has substantial long-term implications including alloimmunization.

From a clinical standpoint, reducing blood product utilization has important implications for resource utilization, cost containment, and patient outcomes. Blood products represent costly and limited resources, and strategies that effectively reduce their use constitute meaningful advances in cardiac surgical care.

The proportion of transfusion-free patients varied considerably across studies, reflecting differences in transfusion protocols and patient populations. Kimenai et al. [11] reported the highest rates of transfusion-free patients (approximately 60–62 % across all groups), likely attributable to their stringent point-of-care transfusion protocol. In contrast, Spegar et al. [12] reported that only 27 % of patients remained transfusion-free, reflecting their complex valve procedures and older patient population. The pediatric cyanotic surgery study by Patel et al. [14] demonstrated a clear advantage for combined therapy, with the lowest blood product requirements in this group.

Clinically, the ability to avoid transfusion altogether represents a significant outcome, as it eliminates transfusion-related complications including immunologic reactions, infection transmission, and circulatory overload. Mortality rates remained consistently low across all studies, with no significant differences between TXA administration strategies.

Mahaffey et al. [13] reported mortality of 1.3 % in the IV-only group versus 0 % in the combined group. Spegar et al. [12] observed a 4 % overall mortality rate equally distributed between groups. Kimenai et al. [11] demonstrated in-hospital mortality rates of 0.8 % (TA group), 1.6 % (placebo group), and 0.8 % (control group) without statistical significance (p = 0.620). Taksaudom et al. [10] and Patel et al. [14] reported no deaths in their respective studies. The absence of mortality differences suggests that different approaches to TXA administration are similarly safe from a survival perspective. No significant increases in venous thromboembolism, infection, or other adverse outcomes were observed following combined TXA use, indicating a favorable safety profile. This is clinically reassuring, particularly given theoretical concerns about systemic absorption and potential thrombotic complications with higher TXA doses.

While combined TXA administration significantly reduced total blood loss, the magnitude of this reduction (∼40 mL) did not translate into clinically relevant outcomes such as transfusion requirements and mortality was not significant. Surgical re-exploration rates due to major bleeding and surrogate endpoints such as intensive care unit and hospital lengths of stay were not significantly different between approaches in any included study.

### 4.1 Study strength and limitations

This study is the most comprehensive synthesis to date on the effects of a combined TXA hemostatic regimen in cardiac surgery. The analysis drew from studies of moderate to high methodological quality, and low statistical heterogeneity across most outcomes strengthens the reliability of the pooled estimates. The main exception was cumulative blood loss, which showed significant heterogeneity.

Nonetheless, several limitations must be considered. A primary issue is that most studies had small sample sizes and were underpowered to detect meaningful differences in clinical events like transfusion needs or adverse effects. The inclusion of one disproportionately large study (Kimenai et al.) may have also skewed the overall results. Further limitations include significant heterogeneity in patient populations, baseline risks, study protocols, and endpoint definitions. In particular, variations in TXA dosing regimens and institutional transfusion protocols make it difficult to compare efficacy and blood product utilization directly across studies [[16], [17], [18], [19]].

Moreover, no formal subgroup analyses, such as adult versus pediatric cohorts, CABG-only versus mixed procedures, or on-pump–only populations, could be robustly performed given the limited number of available studies. This gap highlights the need for future trials specifically designed to address clearly defined high-risk subgroups.

Looking forward, research should aim to identify patient subgroups that benefit most from this combined therapy, paving the way for more personalized bleeding management strategies. Comprehensive cost-effectiveness analyses are also needed to guide clinical and policy decisions [16,17]. Finally, mechanistic studies of the pericardial space could clarify the topical action of TXA, helping to optimize future dosing and delivery strategies.

## 5 Conclusion

The combined use of IV and topical TXA was associated with a statistically significant reduction in cumulative blood loss compared to IV administration alone. However, the modest magnitude of this reduction did not translate into clinically relevant outcomes, as neither transfusion requirements nor mortality were significantly different.

## Data availability statement

The data underlying this article are available in the article and in its online supplementary material.

## CRediT authorship contribution statement

**Paul C. Onyeji:** Writing – original draft, Methodology, Formal analysis, Data curation, Conceptualization. **Leo Consoli:** Visualization, Software, Investigation, Formal analysis, Data curation. **Amrinder Kaur:** Writing – original draft, Visualization, Methodology. **Shivank Dani:** Writing – original draft, Visualization, Investigation. **Sonise Momplaisir-Onyeji:** Visualization, Validation, Data curation, Conceptualization. **Felipe S. Passos:** Writing – review & editing, Validation, Supervision. **Hristo Kirov:** Writing – review & editing, Validation, Supervision. **Torsten Doenst:** Writing – review & editing, Supervision. **Tulio Caldonazo:** Writing – review & editing, Resources, Project administration, Funding acquisition.

## Funding

TC was funded by the Deutsche Forschungsgemeinschaft (DFG, German Research Foundation) Clinician Scientist Program OrganAge funding number 413668513, by the Deutsche Herzstiftung (DHS, German Heart Foundation) funding number S/03/23 and by the Interdisciplinary Center of Clinical Research of the Medical Faculty Jena.

## Declaration of competing interest

The authors declare that they have no known competing financial interests or personal relationships that could have appeared to influence the work reported in this paper.
