# Supplementary material for: Combining topical and intravenous tranexamic acid in cardiac surgery: does it really matter? – a systematic review and meta-analysis
Source: Int J Cardiol Heart Vasc. 2025 Dec 1;62:101848. doi: 10.1016/j.ijcha.2025.101848 (PMC12719039; doi:10.1016/j.ijcha.2025.101848)

**Supplementary Material:**

**Supplementary Table S1.** Complete search strategy.

**Supplementary Figure S1.** Leave-One-Out sensitivity analysis.

**Supplementary Figure S2.** Funnel plot for publication bias assessment.

**Supplementary Figure S3.** Risk of bias assessment. A) Result of ROBINS-I tool for the observational sty. B) Results of the RoB-2 tool for randomized controlled trials.

**Supplementary Table S1.** Complete search strategy

| **Searched databases** | **Search Strategy** |
| --- | --- |
| **Ovid MEDLINE** | (Coronary Surgery OR Cardiac Surgery OR Heart Surgery) AND (Topical Tranexamic acid OR Intravenous Tranexamic acid) |
| **EMBASE** | ('cardiac surgery'/exp OR 'heart surgery'/exp OR 'coronary surgery':ti,ab,kw OR 'cardiac surgery':ti,ab,kw OR 'heart surgery':ti,ab,kw) AND ('topical tranexamic acid':ti,ab,kw OR 'intravenous tranexamic acid':ti,ab,kw) |
| **Cochrane Library** | ("coronary surgery":ti,ab,kw OR "cardiac surgery":ti,ab,kw OR "heart surgery":ti,ab,kw) AND ("topical tranexamic acid":ti,ab,kw OR "intravenous tranexamic acid":ti,ab,kw) |

**Supplementary Figure S1:** Leave-One-Out Sensitivity Analysis for the main outcome of cumulative blood loss. When the study by Patel was removed, statistical significance was lost. **CI:** confidence interval; **MD:** mean difference.


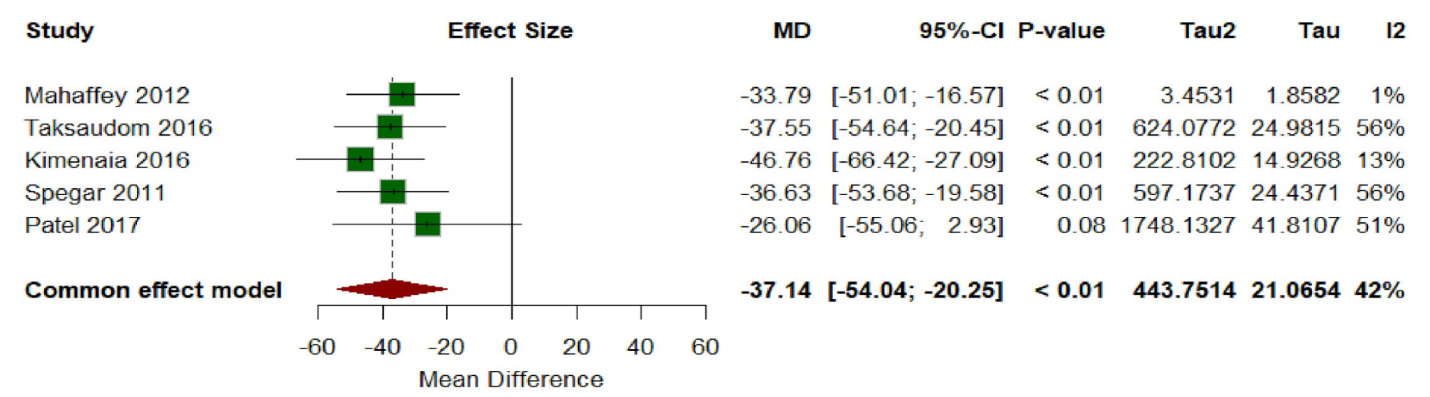


**Supplementary Figure S2**: Funnel Plot for Publication Bias Assessment of the main outcome of cumulative blood loss. No asymmetry was detected by visual inspection.


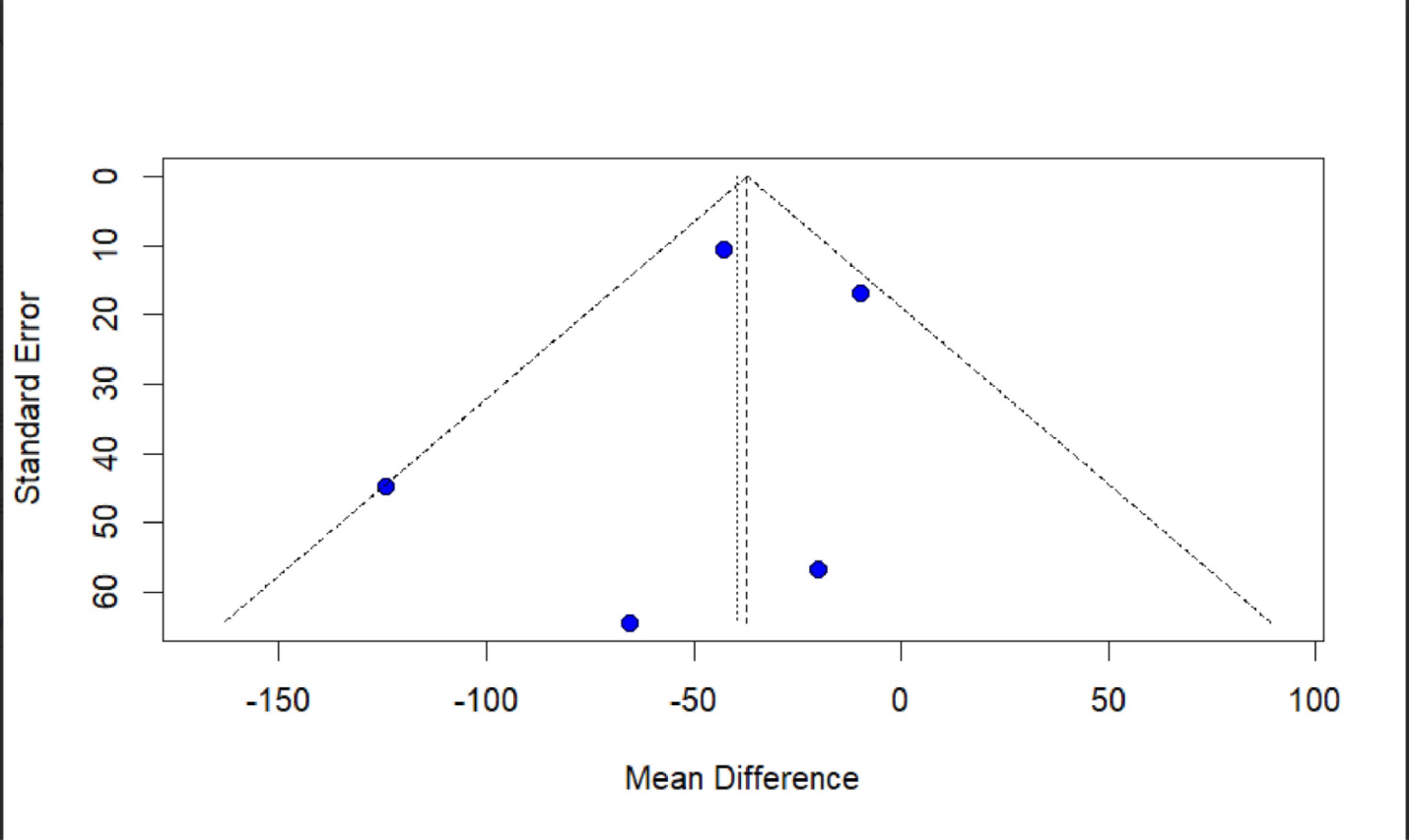


**Supplementary Figure S3**: Results of the risk of bias assessment for observational cohort studies (A) and randomized controlled trials (B).

A


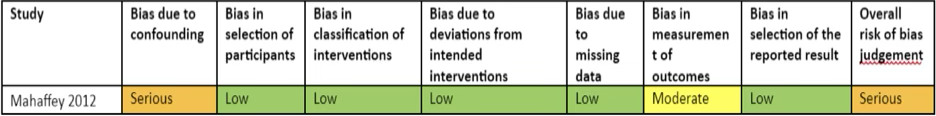


B


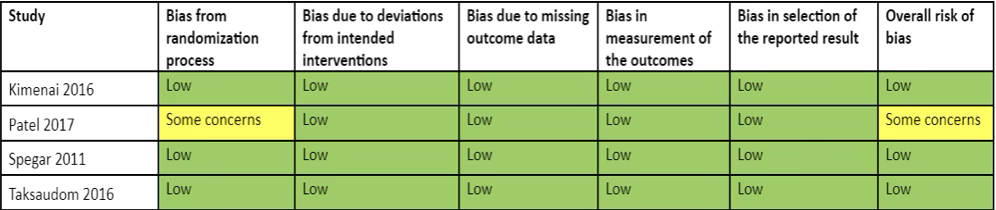

Supplement: Supplementary Data 1 [file mmc1.docx]
